# Stereotactic radiosurgery versus whole-brain radiotherapy after intracranial metastasis resection: a systematic review and meta-analysis

**DOI:** 10.1186/s13014-017-0840-x

**Published:** 2017-06-24

**Authors:** Nayan Lamba, Ivo S. Muskens, Aislyn C. DiRisio, Louise Meijer, Vanessa Briceno, Heba Edrees, Bilal Aslam, Sadia Minhas, Joost J. C. Verhoeff, Catharina E. Kleynen, Timothy R. Smith, Rania A. Mekary, Marike L. Broekman

**Affiliations:** 1 Cushing Neurosurgery Outcomes Center (CNOC), Department of Neurosurgery, Brigham and Women’s Hospital, Harvard Medical School, Boston, MA USA; 2 Harvard Medical School, Boston, MA USA; 3 Department of Neurosurgery, Brain Center Rudolf Magnus, University Medical Center Utrecht, HP G03.124, PO Box 85500, 3508 GA Utrecht, The Netherlands; 4 School of Pharmacy, MCPHS University, Boston, MA USA; 5 Department of Radiation Oncology, University Medical Center Utrecht, Utrecht, The Netherlands

**Keywords:** Brain Metastasis, Resection, Whole brain radiation, Stereotactic radiosurgery, Meta-analysis

## Abstract

**Background:**

In patients with one to three brain metastases who undergo resection, options for post-operative treatments include whole-brain radiotherapy (WBRT) or stereotactic radiosurgery (SRS) of the resection cavity. In this meta-analysis, we sought to compare the efficacy of each post-operative radiation modality with respect to tumor recurrence and survival.

**Methods:**

Pubmed, Embase and Cochrane databases were searched through June 2016 for cohort studies reporting outcomes of SRS or WBRT after metastasis resection. Pooled effect estimates were calculated using fixed-effect and random-effect models for local recurrence, distant recurrence, and overall survival.

**Results:**

Eight retrospective cohort studies with 646 patients (238 with SRS versus 408 with WBRT) were included in the analysis. Comparing SRS to WBRT, the overall crude risk ratio using the fixed-effect model was 0.59 for local recurrence (95%-CI: 0.32–1.09, I^2^: 3.35%, P-heterogeneity = 0.36, 3 studies), 1.09 for distant recurrence (95%-CI: 0.74–1.60, I^2^: 50.5%, P-heterogeneity = 0.13; 3 studies), and 2.99 for leptomeningeal disease (95% CI 1.55–5.76; I^2^: 14.4% p-heterogeneity: 0.28; 2 studies). For the same comparison, the risk ratio for median overall survival was 0.47 (95% CI: 0.41–0.54; I^2^: 79.1%, P-heterogeneity < 0.01; 4 studies) in a fixed-effect model, but was no longer significant (0.63; 95%-CI: 0.40–1.00) in a random-effect model. SRS was associated with a lower risk of leukoencephalopathy (RR: 0.15, 95% CI: 0.07–0.33, 1 study), yet with a higher risk of radiation-necrosis (RR: 19.4, 95% CI: 1.21–310, 1 study).

**Conclusion:**

Based on retrospective cohort studies, the results of this study suggest that SRS of the resection cavity may offer comparable survival and similar local and distant control as adjuvant WBRT, yet may be associated with a higher risk for developing leptomeningeal disease. Future research on SRS should focus on achieving a better understanding of the various factors that may favor SRS over WBRT.

## Background

Brain metastases are an increasingly common complication of systemic cancers and represent a significant source of morbidity and mortality in cancer patients [1–5]. Approximately, 20–40% of cancer patients with primary extracranial cancer will develop brain metastases during the course of their disease [6]. In the United States alone, this represents about 98,000 to 170,000 new diagnoses each year [6, 7]. Median survival without treatment is estimated at 1 month, and increases to 3–12 months when cranial radiation therapy is used [8].

Traditionally, the standard of care for patients with solitary brain metastasis has been resection plus whole-brain radiotherapy (WBRT) [4, 7]. Resection allows for histopathological examination of the tissue and has been shown to improve neurological symptoms, functional independence, and survival [5]. Moreover, several studies found that when resection was followed by adjuvant WBRT, it resulted in improved intracranial tumor control and lower rates of neurologic deaths compared to resection alone [9–13]. One randomized controlled trial (RCT) assigned patients to WBRT or observation following either initial surgery or SRS. While the researchers did not find the addition of WBRT to affect overall survival when compared to observation, they did find that following either surgery or SRS, the addition of WBRT reduced the 2-year relapse rate when compared to surgery without adjuvant WBRT. More specifically, WBRT reduced the probability of relapse from 59 to 27% following surgery. A similar trend was noted following SRS, in which adjuvant WBRT reduced the probability of relapse from 31 to 19%. While the former result is consistent with what other experiments have also demonstrated (i.e. improved disease control with adjuvant WBRT following resection), the latter result is novel in that it suggests a possible role for adjuvant SRS in the management of brain metastases [13]. The fact that SRS followed by WBRT offered better control than SRS alone, along with the fact that surgery plus WBRT led to improved outcomes as compared to surgery alone, suggests the possibility that SRS following surgery may offer similarly improved rates of disease control without the sequelae associated with radiation exposure to the entire brain.

Furthermore, WBRT is associated with both short and long-term neurological complications, such as radiation-induced edema, leukoencephalopathy, and cognitive deficits [2, 7, 10]. Given that patients with systemic cancer are now living longer due to improved extracranial anti-tumor strategies, concerns related to these delayed, radiation-induced neurotoxic effects are prompting neuro-oncologists to assess alternatives to WBRT [4, 14]. Many centers are therefore using targeted techniques with limited radiation exposure, such as stereotactic radiosurgery (SRS). SRS is associated with fewer global cognitive side effects, CNS preservation, and better quality-of-life [2, 4, 7, 15].

In this study, we used the current published literature to compare local recurrence, distant recurrence, leptomeningeal disease, and overall patient survival amongst patients who underwent resection plus SRS to patients who underwent resection plus WBRT for treatment of one to three intracranial metastases. Given the available data and abovementioned RCT, we hypothesize that SRS may lead to similar rates of local control as compared to WBRT following resection, but, based upon the targeted scope of SRS, may lead to poorer rates of distant control and overall patient survival.

## Methods

This meta-analysis was done in accordance with the Preferred Reporting Items for Systematic Reviews and Meta-Analyses (PRISMA) statement [16]. PubMed, EMBASE, and Cochrane databases were searched on 6-25-2016 for studies comparing outcomes of intracranial metastasis resection followed by WBRT versus intracranial metastasis resection followed by SRS using the following keywords: Whole-Brain Radiotherapy, Stereotactic Radiosurgery, resection and intracranial metastasis with synonyms (Appendix 1). Language was limited to English.

### Eligibility criteria and study selection

Inclusion criteria consisted of any study that described adult patients (≥18 years of age) with a diagnosis of systemic cancer who underwent neurosurgery followed by whole-brain radiotherapy (WBRT) or by stereotactic radiosurgery (SRS) for treatment of up to three metastases to the brain. SRS was defined as a single or few fractions of high dose radiation to a small intracranial target that was encompassed by at least 50% of the prescribed dose [17]. Because Intraoperative Radiation Therapy (IORT) meets most of the salient features of SRS as described by the Radiation Oncology Therapy Group protocol, [18, 19] studies reporting IORT were included within the SRS treatment groups. Studies reporting on the use of Local Brain Radiotherapy (LBRT), however, were excluded for not meeting the criteria of SRS. Studies reporting outcomes on patients receiving prior WBRT and studies that did not compare post-operative WBRT to post-operative SRS were also excluded. After title and abstract screening, remaining articles were read full-text. Three authors (NL, IM, and LM) performed full-text screening and two (NL and LM) extracted the data. One senior author (MB) reviewed the included articles and the extracted data. Disagreements were solved by discussion.

### Data extraction and management

The following data were extracted from each study whenever possible: author and year of published articles, number of patients in the study, patient characteristics (inclusion criteria, primary tumor type, age, type of intervention (whole-brain radiotherapy, stereotactic radiosurgery, radiation dose and regimen, time between resection and radiation), number of intracranial lesions at presentation, extent of extracranial disease), duration of follow-up, and primary and secondary outcomes of the study. When available, relative risks (RR) comparing SRS to WBRT were extracted for local recurrence risk, distant recurrence, leptomeningeal disease, and overall survival. Duration of overall survival (OS) and progression free survival (PFS) for both local and distant recurrence was also noted. Furthermore, data on the number of patients that died from neurological causes and on observed neurotoxicity were extracted. In one study reporting outcomes of multiple metastases per patient, the reported percent recurrence by number of cavities was considered as equivalent to the percent recurrence by number of patients, as the number of metastases per patient approximated 1:1 [20].

Study quality was assessed by the New-Castle Ottawa Scale, and the quality of our recommendation was assessed using the Grading of Recommendations, Assessment, Development and Evaluations (GRADE) criteria. Both assessments were made by two authors (NL and IM) independently [21, 22]. Discrepancies were solved by discussion.

### Data analysis

To minimize heterogeneity amongst studies being compared, only cohort studies were included in the meta-analysis. Data analysis was performed using Comprehensive Meta-Analysis (CMA) Version 3 (Biostat, Inc., Englewood, NJ, USA). Overall fixed and random risk ratios were calculated for the same outcomes, depending on the available data in the articles. I-squared values were calculated to assess heterogeneity. For median overall survival, relative risks were calculated based on group size and median overall survival both for SRS and WBRT [23]. Meta-regression analysis on continent, age, and journal impact-factor was used when possible to assess sources of heterogeneity.

## Results

**Fig. 1:**
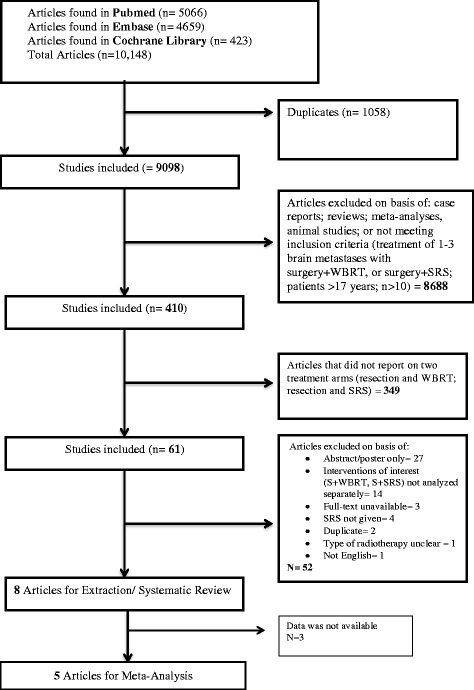
Study selection process of the included articles

The search strategy resulted in 9908 articles after removal of duplicates. After screening for titles and abstracts, 410 full-texts were screened. Eight retrospective cohort studies provided outcomes for 646 patients treated with WBRT (n = 408) or SRS (n = 238) following resection (Fig. 1) [5, 18, 20, 24–28]. The most common primary tumor histologies were non-small cell lung cancer and colon cancer. Mean group size was 30 patients for SRS and 51 for WBRT. The mean age for the SRS-treated groups was 58 while the mean age for the WBRT-treated groups was 56 years. Mean radiation dose was single dose 18 Gy for SRS and fractionated dose 35 Gy for WBRT. The Newcastle-Ottawa Scale (NOS) varied between 6 and 8 among the studies (Table 1) and all studies were deemed of very low quality based on GRADE criteria (Table 2).

**Table 1:** Characteristics of the studies included in the systematic review (n = 8) and meta-analysis (*n* = 6)

Author (year of publication)	Total Number of patients	Treatment modality (number of patients)	Number of Cases (number of metastases)	Mean Age	Pathology of the metastasis								Radiation dose (Scheme)	Radiation dose (EQD2)	NOS score
					Lung (SC)	Lung (NSC)	Melanoma	Colon	Renal	Breast	Other	Unknown			
Elaimy (2011) ^a^ ^16^	275	Resection + WBRT (11 patients)	7 patients (1 metastasis) 4 patients (unknown)	60	2	6	1	0	UA	0	0	2	Median: 30 Gy (NA)	37.5 Gy	7
		Resection + SRS (15 patients)	10 patients (1 metastasis) 3 patients (2–4 metastases)	57	1	6	4	UA	2	0	2	0	Median: 18 Gy (1 dose)	90.0 Gy	
Gu (2015)^17^	93	Resection + WBRT (19 patients)	37 patients (1 metastasis) 8 patients (2–3 metastases)	UA	0	0	0	19	0	0	0	0	30 Gy (10x3Gy)	37.5 Gy	6
													40 Gy (20x2 Gy)	40.0 Gy	
		Resection + SRS (11 patients)		UA	0	0	0	11	0	0	0	0	12–24 Gy (1 dose)	42.0−> 110.0Gy	
Hsieh (2015)^18^	212	Resection + WBRT (156 patients)	83 patients (1 metastasis) 52 patients (2–3 metastases)	58	UA	78	17	UA	UA	24	31	UA	30 Gy (10 x 3 Gy)	37.5 Gy	7
													37.5 Gy (15x2.5 Gy)	42.2 Gy	
		Resection + SRS (56 patients)	42 patients (1 metastasis) 14 patients (2–3 metastases)	60	UA	30	5	UA	UA	6	14	UA	18 Gy (1 dose)	56.0−> 110.0 Gy	
Hwang (2010) ^a19^	43	Resection + WBRT (18 patients)	1.67 +/− 0.91 ^b^	52.8	1	12	UA	UA	UA	1	2	2	UA	UA	7
		Resection + SRS (25 patients)	1.54 +/− 0.72 ^b^	59.47	1	15	UA	UA	UA	4	3	0	UA	UA	
Lee (2013) ^a5^	157	Resection + WBRT (109 patients)	96 patients (1 metastasis) 61 patients (2–3 metastases)	UA	UA	UA	UA	UA	UA	UA	UA	UA	30 Gy (10x3 Gy)	37.5 Gy	7
													40 Gy (20x2 Gy)	40.0 Gy	
		Resection + SRS (17 patients)		UA	UA	UA	UA	UA	UA	UA	UA	UA	15–24 Gy (1 dose)	63.8−> 110 Gy	
Patel (2014)^24^	132	Resection + WBRT (36 patients)	14 patients (1 metastasis) 22 patients (2–3 metastases)	54.6	UA	15	UA	UA	UA	UA	21?	UA	30 Gy (10x3Gy)	37.5 Gy	8
													37.5 Gy	42.2 Gy	
		Resection + SRS (96 patients)	68 patients (1 metastasis) 28 patients (2–3 metastases)	56	0	45	UA	UA	UA	UA	51	UA	15–21Gy (1 dose)	63.8−> 110.0 Gy	
Salvati (1997) ^a28^	19	Resection + WBRT (14 patients)	14 patients (1 metastasis)	UA	0	0	14	0	0	0	0	0	40–50Gy (NA)	40.0–50.0 Gy	6
		Resection + SRS (5 patients)	5 patients (1 metastasis)	UA	0	0	5	0	0	0	0	0	UA	UA	
Caroli (2011)^29^	204	Resection + WBRT (45 patients)	Not available	UA	UA	UA	UA	UA	UA	UA	UA	UA	UA	UA	7
		Resection + SRS (13 patients)		UA	UA	UA	UA	UA	UA	UA	UA	UA	UA	UA	

*Abbreviations*: *SRS* Stereotactic Radiosurgery, *WBRT* Whole Brain Radiotherapy, *SC* Small-cell, *NSC* Non-Small Cell, *Gy* Gray, *NOS* New-Castle Ottawa, *UA* Unavailable, *EQD2* equivalent dose in 2-Gy fractions (EQD2(α/β = 2)), *RCS* Retrospective cohort study

^a^ included in the meta-analysis

^b^ reported as mean +/− SD

**Table 2:** The Grading of Recommendations, Assessment, Development and Evaluations (GRADE) Criteria was used to assess the level of evidence for each outcome

Outcomes	Type of Evidence	Quality	Consistency	Directness	Effect Size	Total	Overall Quality
Local Control	+2	−2	0	0	0	0	Very low
Distant Progression	+2	−2	0	0	0	0	Very low
LMD	+2	−2	−1	0	+1	0	Very low
Overall Survival	+2	−2	−1	0	0	−1	Very low

Type of evidence is based on the study design of the included studies and ranges from +2 to +4. Study quality is graded based on blinding and allocation, follow up and withdrawals, sparsity of data, or methodological concerns and ranges from −3 to 0. Consistency is graded based on heterogeneity of populations and study end points with respect to one another and included populations and ranges from −1 to +1. Directness is graded based on generalizability of the included results and is graded from −2 to 0. Effect size is graded on the value of the RR or OR and is graded from 0 to +2. The overall quality of the recommendations that can be made based on the included studies includes the following categories: high (at least 4 points overall), moderate (3 points), low (2 points), and very low (1 point or less)

### Local recurrence

Data regarding local recurrence was available in five out of the eight studies included in our review [5, 18, 20, 26, 27]. The local recurrence incidence ranged from 0 to 60% in patients treated with adjuvant SRS and from 11 to 24% in patients treated with adjuvant WBRT. While no study reported a statistically significant difference in local recurrence rate between the two treatment groups, four of the five studies that included data on local recurrence did note differences in these rates. One study reported a near-significant hazard ratio (*p* = 0.09) that disfavored SRS over WBRT by a 68% higher rate of distant recurrence [18]. One Italian study from 1996 that did not specifically report a *p*-value comparing just these two treatment arms did find that 5 patients treated with adjuvant SRS were nearly four times as likely to experience local recurrence as compared to 14 patients treated with adjuvant WBRT [27]. Two other studies favored SRS for achieving local control, with percent differences in local recurrence ranging from 3 to 17% in favor of SRS [20, 26]. Only the study by Lee et al. demonstrated near-equal rates of local recurrence for both treatment groups, with a local recurrence of 11 and 12% for WBRT and SRS treatment groups, respectively [5].

**Fig. 2:**
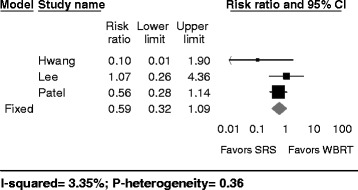
Local recurrence after SRS versus WBRT following neurosurgical resection. Forest plot represents the risk ratio for local recurrence of intracranial metastasis following resection and SRS versus resection and WBRT (95% confidence interval [CI]) with 5 cohort studies in adults (*n* = 138 patients (SRS) and 163 patients (WBRT)). *Solid squares* represent the point estimate of each study and the *diamond* represents the pooled estimate of the risk ratio. The *I* ^2^ and *P* values for heterogeneity are shown

The pooled risk ratio comparing local recurrence incidence between SRS vs. WBRT groups was 0.79 (95% CI: 0.48–1.29; fixed effects model) and demonstrated no significant difference in rates of local recurrence (Fig. 2). Results from the random effects model were similar (RR: 0.88; 95%-CI = 0.40–1.92) and moderate heterogeneity was observed (I^2^ = 49.44%, p-heterogeneity = 0.10, 3 studies). This analysis was based on 163 patients receiving post-operative WBRT and 138 patients receiving postoperative SRS.

Univariate meta-regression on age (*p* = 0.02) and continent (*p* = 0.04) were identified as a source of heterogeneity. With increasing age, the RR for local recurrence was in favor of SRS as compared to WBRT. Furthermore, Europe, in comparison to North America was associated with a RR for local recurrence in favor of WBRT. Journal impact factor was not identified as a source of heterogeneity (*p* = 0.97).

### Distant progression

**Fig. 3:**
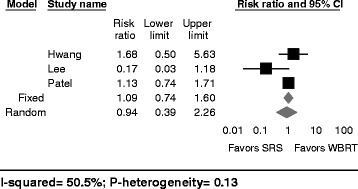
Distant progression after SRS versus WBRT following neurosurgical resection. Forest plot represents the risk ratio for distant recurrence of intracranial metastasis following resection and SRS versus resection and WBRT (95% confidence interval [CI]) with 4 cohort studies in adults (*n* = 138 patients (SRS) and 163 patients (WBRT)). *Solid squares* represent the point estimate of each study and the *diamond* represents the pooled estimate of the risk ratio. The *I* ^2^ and *P* values for heterogeneity are shown

Data regarding distant progression was available in three out of the eight studies included in our study (Fig. 3) [5, 20, 26]. The distant progression incidence ranged from 6 to 50% in patients treated with adjuvant SRS and from 17 to 44% in patients treated with adjuvant WBRT [5, 20, 26]. Three of the studies, while not reaching statistical significance, also reported distant progression rates that were 6 to 11% higher in SRS patients as compared to WBRT patients [20, 26]. One study reported better distant control in 17 patients treated with SRS as compared to 109 treated with WBRT [5]. One additional study reported a statistically significant distant progression hazard ratio of 2.17 for patients treated with SRS (single dose range: 14 to 24 Gy) after resection compared to those treated with WBRT (dose: 30 Gy in 10 fractions or 37.5 Gy in 15 fractions) [18]. The pooled risk ratio comparing distant recurrence rates between SRS versus WBRT was 1.09 (95%-CI: 0.74–1.60; fixed effects model) and demonstrated no significant difference in rates of distant recurrence (Fig. 3). Results from the random effects model were similar (RR: 0.94; 95%-CI: 0.39–2.26), and moderate heterogeneity was observed (I^2^ = 50.5%, p-heterogeneity = 0.28, 3 studies, Fig. 3). Continent (*p* = 0.52), age (*p* = 0.12), and journal impact factor (*p* = 0.52) were not identified as sources of heterogeneity. This analysis was based on 163 patients receiving postoperative WBRT and 138 patients receiving postoperative SRS.

### Leptomeningeal disease

Two studies reported on the development of leptomeningeal disease (LMD) following radiotherapy [18, 20]. Retrospectively, SRS treatment relative to WBRT was associated with a higher risk of LMD occurrence as per Hsieh et al. [18] (HR:2.44; 95%-CI:1.15–5.18) and Patel et al. [24] (HR:5.67; 95%-CI: 1.50–21.51).

**Fig. 4:**
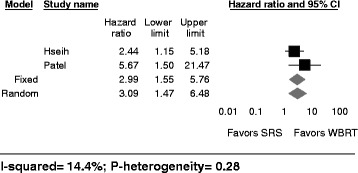
Leptomeningeal Disease after SRS versus WBRT following neurosurgical resection. Forest plot represents the risk ratio for overall survival of intracranial metastasis following resection and SRS versus resection and WBRT (95% confidence interval [CI]) with 2 cohort studies in adults (*n* = 152 patients (SRS) and 192 patients (WBRT)). *Solid squares* represent the point estimate of each study and the *diamond* represents the pooled estimate of the risk ratio. The *I* ^2^ and *P* values for heterogeneity are shown

The pooled relative risk for the development of LMD following postoperative SRS to postoperative WBRT was 2.99 (95% CI 1.55–5.76), indicating that resection followed by SRS increases the risk for developing LMD as compared to resection followed by WBRT (Fig. 4). There was low heterogeneity (I^2^: 14.4% p-heterogeneity: 0.28, 2 studies) between the included studies. This remained significant in the random effects model (RR: 3.09, 95% CI: 1.47–6.48). The analysis is based on 192 patients receiving resection followed by WBRT and 152 patients receiving resection followed by SRS.

### Overall survival

Only one study out of the 8 included in this review found a significant difference in survival between patients treated with SRS following resection as opposed to WBRT [24]. In their 2011 institutional study, Elaimy et al. retrospectively compared six different treatment regimens in patients with metastatic lesions in the brain [24]. Using SRS only as a reference, they found that 15 selected patients treated with SRS following resection had a 68% lower risk of death as compared to 65 patients treated with SRS alone (*p* = 0.02). In contrast, they reported a slightly higher risk of death (HR = 1.04) in 11 patients treated with WBRT after resection as compared to SRS alone [24]. While the other eight studies did not report significant differences in survival between the two treatment groups, two studies were slightly in favor of SRS, [18, 26] while three [5, 27, 28] indicated slightly better outcomes after WBRT, with one even reaching a near-significant level in favor of WBRT (*p* = 0.06) [5].

**Fig. 5:**
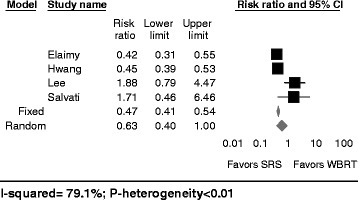
Survival after SRS versus WBRT following neurosurgical resection. Forest plot represents the risk ratio for overall survival of intracranial metastasis following resection and SRS versus resection and WBRT (95% confidence interval [CI]) with 4 cohort studies in adults (*n* = 153 patients (SRS) and 174 patients (WBRT)). *Solid squares* represent the point estimate of each study and the *diamond* represents the pooled estimate of the risk ratio. The *I* ^2^ and *P* values for heterogeneity are shown

The pooled relative risk for median overall survival comparing patients treated with SRS after resection to those treated with WBRT was 0.51 (95%-CI: 0.44–0.54; I^2^: 86.6%, p-heterogeneity < 0.01; 4 studies), suggesting a favorable outcome for WBRT with considerable heterogeneity (Fig. 5) [5, 24, 26, 27]. However, the overall survival was non-significant using the random-effect model (RR: 0.78; 95%-CI: 0.40–1.00) [5, 24, 26, 27]. The analysis was based on 174 patients receiving post-operative WBRT and 153 patients receiving post-operative SRS. Univariate meta-regression on continent (*p* < 0.01) and journal impact factor (*p* < 0.01) were identified as sources of heterogeneity. Europe and Asia, in comparison to North America, were associated with a RR for overall survival in favor of SRS. A lower impact factor journal showed a RR in favor of WBRT as compared to SRS. Age was not identified as a source of heterogeneity (*p* = 0.14).

### Radiation necrosis and leukoencephalopathy

Radiation necrosis and leukoencephalopathy were each reported in one study. Patel et al. found that symptomatic radiation necrosis, requiring the use of steroid or other interventions, was 27% in the SRS group and 0% in the WBRT group (*p* < 0.01). After 1 year, the rate of radiographic leukoencephalopathy was 7% following SRS vs. 47% following WBRT (*p* < 0.01). The relative risk of leukoencephalopathy for SRS compared to WBRT in this study was 0.15 (95% CI: 0.11–0.21).

## Discussion

The present study demonstrated that there was no statistically significant difference in local recurrence or distant recurrence between patients receiving SRS versus WBRT following neurosurgical resection of brain metastases in retrospective cohorts. Of note, there was a significant increased risk for the development of LMD in patients who received postoperative SRS as compared to WBRT, and a possible survival benefit to WBRT shown only in the fixed effects model with considerable heterogeneity. Though there was little published on leukoencephalopathy and radiation necrosis in these patients, it appeared that SRS reduced the risk of leukoencephalopathy and increased the risk for radiation necrosis as compared to WBRT. These results were in support of our hypothesis regarding similar rates of local control between the two treatment arms, but against our hypothesis predicting that SRS would lead to poorer rates of distant control. The possible survival benefit with WBRT in the fixed effects model was consistent with our hypothesis.

No randomized controlled trials have yet been completed for post-operative radiation modalities. However, a meta-analysis on the effect of the addition of WBRT to SRS or surgery did show less intracranial disease progression, but no effect on overall survival [29]. This is in line with the results of a trial evaluating additional WBRT to SRS that found improved intracranial tumor control but no effect on survival [15]. However, the addition of SRS to WBRT was found to result in longer survival in a randomized trial [30]. A systematic review evaluating linac-based SRS to resection cavities concluded that this treatment strategy provided good local control rates but poor distant intracranial control. This is different form our study that focused on a comparison between SRS and WBRT after resection [31].

Other studies have reported on the high risk of LMD in patients receiving SRS to the resection cavity, and have cited incidence rates around 12–14% [32–34]. Breast cancer metastases have been shown to have a particularly high rate of LMD [32–34]. Additionally, many studies reported on the adverse side effects of WBRT, including physical symptoms such as alopecia, somnolence, hearing loss, skin changes, and neurocognitive decline, manifested as memory loss and learning impairment [2, 7, 18, 26]. In particular, the cognitive decline following WBRT has been linked to leukoencephalopathy, which is supported by the increased risk for leukoencephalopathy in patients receiving post-operative WBRT [20, 35] Another study, evaluating 59 patients treated with primary SRS, found that even after multiple courses of SRS, quality of life (QOL), measured with the EQ-5D instrument, was preserved in 77% of patients at 12 month follow-up [36]. Thus, considering that our present analysis demonstrates similar results with respect to local recurrence, distant progression, and potentially survival in post-operative SRS patients, SRS is a valid treatment option that can help maintain neurocognition and QOL.

In patients with a limited number of brain metastases receiving resection, the results of this study suggest that it is important to understand the risks and benefits of both WBRT and SRS. The possibility for an improved quality of life should be considered when discussing treatment options. Although there is still not a clear formula to guide use of SRS or WBRT following resection, the fact that SRS may offer similar tumor control as WBRT, with the potential for fewer cognitive side effects and less invasiveness, suggests that SRS should be considered as a valid treatment option for brain metastases [26, 37]. While it has been widely acknowledged that WBRT is associated with cognitive decline, SRS has also been associated with radiation induced necrosis [38]. While these studies showed a potential survival improvement in WBRT as compared to SRS in the random effects model, it is important to note that the fixed effects model was not significant and that the considerable heterogeneity between the studies limits the conclusions that can be drawn from this. Both continent and journal impact factor were identified as significant sources of heterogeneity. Lastly, we demonstrated an increased risk for LMD in patients receiving postoperative SRS to the resection cavity, which is an important outcome to discuss with patients considering SRS.

### Limitations and strengths

A major limitation of this review and meta-analysis was the heterogeneity among the included studies. Amongst the nine studies included, one included only colon cancer patients, one included melanoma patients only, and the remaining six consisted of patient populations with varying proportions of melanoma, colon, renal, breast, and unknown histologies [5, 18, 20, 24, 26, 28]. Additionally, the number of brain metastases was not reported homogenously between studies, and reported outcomes were not specific to the number of brain metastases and treatment modalities. Therefore, it was not possible to perform a subgroup analysis by histology type or number of metastases, or address potential sources of heterogeneity by these covariates. This is most likely of importance, because certain tumors, such as melanoma, colon, and renal carcinomas, are considered radioresistant for WBRT [10, 39, 40] and it is possible that outcomes could differ in patients with only a single metastasis [41, 42]. Of note, one study included in the analysis reported patients with 2–4 metastases in a single group, but noted that the number of metastases did not have an effect on the outcomes [24]. A further limitation of this analysis was that some studies reported death as a hazard ratio, while other used the Kaplan Meier method. Reporting a hazard ratio is a more accurate method and the variation in reporting limits the strength of the conclusions. Lastly, the studies included in the analysis were all retrospective in nature. Therefore, the different treatment-strategies implemented for each set of patients were the result of the tumor boards’ selection of patients whom they felt would benefit from that regimen, resulting in a selection bias. This is further complicated by the different forms of radiation administered in each study, as some studies described outcomes of IORT instead of SRS [18]. No studies were identified that compared WBRT with hypofractionated SRS, which has become a viable treatment strategy for lesions not treatable with a single fraction SRS [43, 44].

Despite these limitations, this meta-analysis based on cohort studies evaluated several different outcomes after extensive review of the literature. To our knowledge, this is the first meta-analysis on this topic. The use of both random- and fixed-effect models together with heterogeneity analysis resulted in a critical evaluation of our outcomes. Furthermore, an increased risk for the development of LMD after SRS compared to WBRT following surgical resection was identified.

### Future directions

There is a clear necessity for the improvement of care for brain metastasis patients as they tend to live longer. Currently, several treatment strategies exist for brain metastasis, but data on the most effective strategy is lacking. Factors such as radiation dose, treatment timing, chemotherapy combinations among others should be evaluated in future studies in order to evaluate the most effective treatment modalities. The wide range of treatment modalities also provides a potential to strive towards more individualized patient care. In addition, evaluated outcomes should not be limited to survival or progression and should include quality of life. Indeed, currently a phase III randomized trial is being conducted to evaluate postoperative SRS versus WBRT in brain metastasis patients (NCT01372774) [45]. Primary endpoints will be evaluation of survival and neurocognitive progression and secondary endpoints will be QOL, adverse events, and functional independence among others. Last, the use of “big data” may provide a method to evaluate these outcomes more thoroughly.

## Conclusions

This meta-analysis suggested that post-operative, localized SRS may offer local protection of a similar degree as post-operative WBRT in patients with one to three lesions. WBRT as compared to SRS, however, seemed to offer better protection from LMD and may offer better protection from distant progression of intracranial metastases with improved survival, albeit not reaching statistical significance in this meta-analysis. However, because WBRT has been consistently associated with post-operative development of physical and cognitive symptoms, SRS is a valid alternative to consider, and can be used in multiple sessions if distant metastases eventually do develop. Notably, the long-term risks of radiation from SRS must also be considered and weighed against the long-term risks of WBRT.

## Appendix 1

Search String. (Search performed on 6-25-16)

**Table:**

PUBMED (5066): (Surg*[Tw] OR resect*[Tw] OR operation*[Tw] OR operativ*[Tw] OR "Surgical Procedures, Operative"[MeSH Terms] OR "Neurosurgical Procedures"[Mesh:NoExp] OR "Neurosurgery"[Mesh] OR "Craniotomy"[Mesh] OR "Neurosurgery"[Mesh] OR "Brain/surgery"[Mesh] OR craniotom*[tw] OR neurosurg*[tw]) AND
(Radiotherap*[Tw] OR Radiosurg*[Tw] OR LINAC[Tw] OR linear accelerator[Tw] OR (Gamma Knife[Tw]) OR Cyberknife[Tw] OR "x knife"[Tw] OR stereotactic*[Tw] OR fraction*[Tw] OR irradiat*[Tw] OR radiat* OR WBRT[Tw] OR SRS[Tw] OR SFRT[Tw] OR Radiotherapy[MeSH Terms] OR Dose fractionation[MeSH Terms] OR Cranial Irradiation[MeSH Terms] OR Radiosurgery[MeSH Terms]) AND ((Brain[Tw] OR "Brain"[MeSH Terms] OR Cerebr*[Tw] OR "Cerebrum"[MeSH Terms] OR Cerebell*[Tw] OR Intracranial[Tw]) AND (Metasta*[Tw] OR Neoplasm Metastasis[MeSH Terms])) Filters: English
Embase (4659): (surg* OR resect* OR operation* OR operativ* OR craniotom* OR neurosurg* OR ‘neurosurgery’/exp OR ‘neurosurgery’ OR ‘brain surgery’/exp OR ‘brain surgery’ OR ‘craniotomy’/exp OR ‘craniotomy’) AND (radiotherp* OR radiosurg* OR linac OR ‘gamma knife’/exp OR ‘gamma knife’ OR ‘cyberknife’/exp OR cyberknife OR ‘x knife’ OR stereotactic OR irradiati* OR radiat* OR wbrt OR srs OR SFRT OR ‘radiotherapy’/exp OR ‘radiotherapy’ OR ‘gamma knife radiosurgery’/exp OR ‘gamma knife radiosurgery’ OR ‘brain radiation’/exp OR ‘brain radiation’ OR fraction* OR ‘radiation dose’/exp OR ‘linear accelerator’/exp OR ‘linear accelerator’ OR ‘stereotactic radiosurgery’/exp OR ‘stereotactic radiosurgery’ OR ‘stereotactic body radiation therapy’/exp OR ‘stereotactic body radiation therapy’) AND ((brain OR cerebr* OR cerebell* OR intracranial OR ‘cerebellum’/exp OR ‘cerebellum’ OR ‘brain’/exp OR ‘brain’ OR ‘brain tumor’/exp OR ‘brain tumor’) AND (metastas* OR ‘brain metastasis’/exp OR ‘brain metastasis’)) ([english]/lim) AND [embase]/lim NOT [medline]/lim Cochrane (432): #1 MeSH descriptor: [Neurosurgery] explode all trees #2 MeSH descriptor: [Surgical Procedures, Operative] explode all trees #3 MeSH descriptor: [Neurosurgical Procedures] this term only #4 MeSH descriptor: [Craniotomy] explode all trees #5 Surg* or resect* or operation* or operativ* or craniotom* or neurosurg #6 #1 or #2 or #3 or #4 or #5 #7 MeSH descriptor: [Radiotherapy] explode all trees #8 MeSH descriptor: [Dose Fractionation] explode all trees #9 MeSH descriptor: [Cranial Irradiation] explode all trees #10 MeSH descriptor: [Radiosurgery] explode all trees #11 Radiotherap* or Radiosurg* or LINAC or "linear accelerator" or "Gamma Knife" or Cyberknife or "x knife" or stereotactic* or fraction* or irradiat* or radiat* or WBRT or SRS or SFRT #12 #7 or #8 or #9 or #10 or #11 #13 Brain or Cerebr* or Cerebell* or Intracranial #14 MeSH descriptor: [Cerebrum] explode all trees #15 MeSH descriptor: [Brain] explode all trees #16 #13 or #14 or #15 #17 metasta* #18 MeSH descriptor: [Neoplasm Metastasis] explode all trees #19 #17 or #18 #20 #19 and #16 #21 #6 and #12 and #20

## Abbreviations

CMA: Comprehensive Meta-Analysis
EQD2: Equivalent dose in 2-Gy fraction
GRADE: Grading of Recommendations, Assessment, Development and Evaluations
Gy: Gray
HR: Hazard ratio
IORT: Intraoperative radiotherapy
LMD: Leptomeningeal disease
MMSE: Mini-Mental State Examination
NOS: Newcastle-Ottawa Scale
NSC: Non-Small Cell
OS: Overall survival
PFS: Progression free survival
PRISMA: Preferred Reporting Items for Systematic Reviews and Meta-Analyses
QOL: Quality of Life
RCS: Retrospective cohort study
RCT: Randomized controlled trial
RR: Risk ratio
SC: Small-cell
SRS: Stereotactic radiosurgery
UA: Unavailable
VMAT: Volumetric Mediated Arc Therapy
WBRT: Whole brain radio therapy
